# Supplementary material for: Time‑resolved multi-omic analysis of paclitaxel exposure in human iPSC‑derived sensory neurons unveils mechanisms of chemotherapy‑induced peripheral neuropathy
Source: Cell Death Dis. 2026 Feb 10;17(1):211. doi: 10.1038/s41419-026-08445-2 (PMC12921266; doi:10.1038/s41419-026-08445-2)
Supplement: Supplementary file 15 — Dataset 1. [file 41419_2026_8445_MOESM15_ESM.zip › CDD_Open_Data/04_RNA_Seq/Timeline_panel_plots_etc_2023-01-23_1609.zip!/Panel_Plots_etc/Evidence_Plots/72h/evidence_plot_standard contrasts_msigdb_reactome_72h_PTX_vs_DMSO_ID0_M27172_REACTOME_ACTIVATION_OF_GENE_EXPRESSION_BY_SREBF_SREBP.pdf]

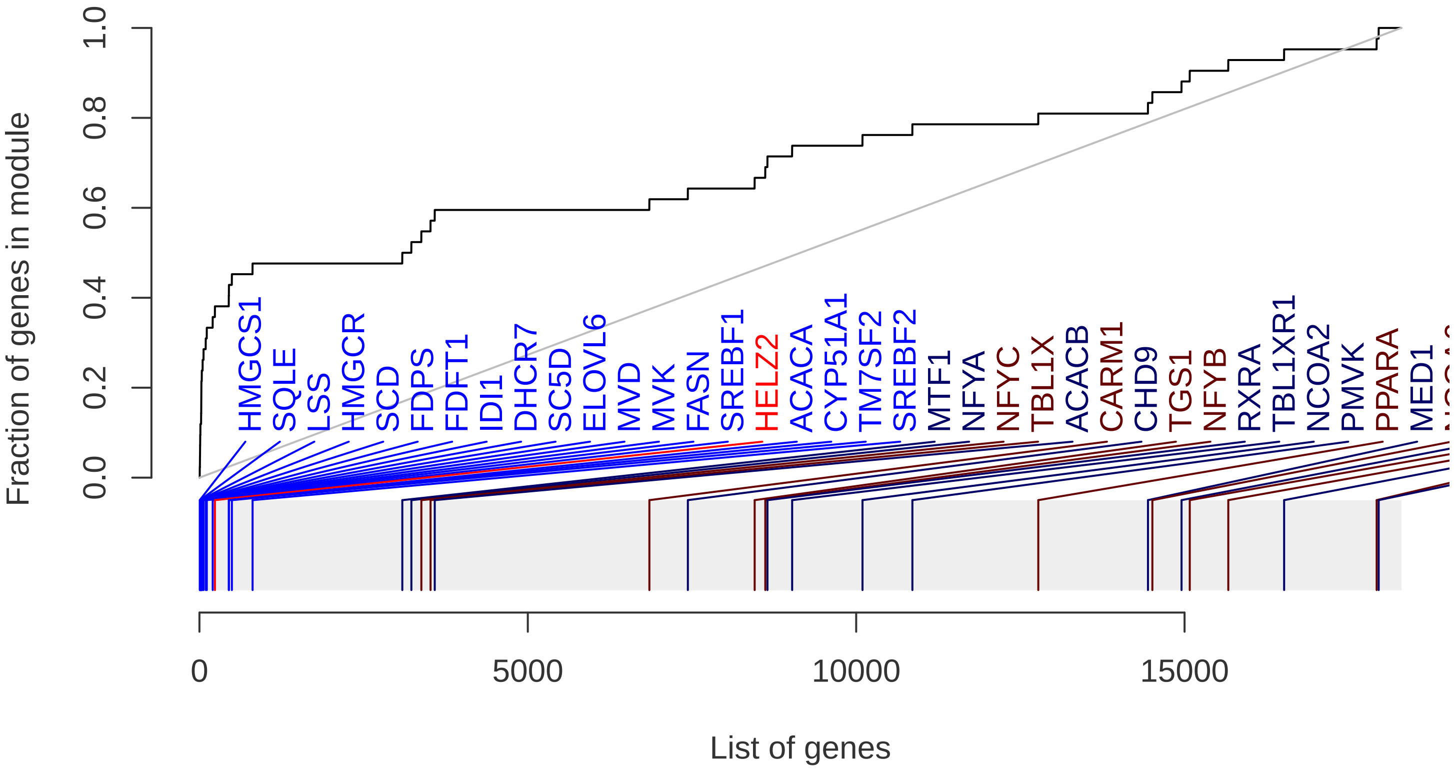

Fraction of genes in module
0.0
0.2
0.4
0.6
0.8
1.0
HMGCS1
SQLE
LSS
HMGCR
SCD
FDPS
FDFT1
IDI1
DHCR7
SC5D
ELOVL6
MVD
MVK
FASN
SREBF1
HELZ2
ACACA
CYP51A1
TM7SF2
SREBF2
MTF1
NFYA
NFYC
TBL1X
ACACB
CARM1
CHD9
TGS1
NFYB
RXRA
TBL1XR1
NCOA2
PMVK
PPARA
MED1
0
5000
10000
15000
List of genes
